# International Federation of Clinical Chemistry (IFCC): Scientific Division, Committee on Apolipoproteins; Working Group of Antibody Reagents: Selection and Characterization of Monoclonal Antibodies for Measuring Plasma Levels of Apolipoproteins A-I and B

**DOI:** 10.1155/S1463924690000256

**Published:** 1990

**Authors:** Santica Marcovina, Linda K. Curtiss, Ross Milne, John J. Albers

**Affiliations:** 1 Northwest Lipid Research Center 2121 N 35th St Seattle Washington 98103 USA; 2 LaJolla USA; 3 Montreal Canada; 4 Seattle USA


Journal of Automatic Chemistry, Vol. 12, No. 5 (September–October 1990), pp. 195–198

### International Federation of Clinical Chemistry (IFCC): Scientific Division, Committee on Apolipoproteins; Working Group of Antibody Reagents: Selection and Characterization of Monoclonal Antibodies for Measuring Plasma Levels of Apolipoproteins A–I and B†

**Prepared for publication by: Santica Marcovina, Seattle, USA (Chairman)*; Linda K. Curtiss, La Jolla, USA; Ross Milne, Montreal, Canada; and John J. Albers, Seattle, USA**

### Introduction

Measurements of apolipoproteins A–I and B are of growing interest in the clinical and research laboratory because levels of these proteins in the blood have been shown to serve as important predictors of atherosclerotic vascular disease, and they provide information not available from total cholesterol or lipoprotein cholesterol levels. Apolipoprotein A–I and B measurements have not reached their full potential in the clinical laboratory because of inadequate standardization and problems in methodology. Although both polyclonal and monoclonal antibodies have been used extensively in apolipoprotein assays, monoclonal antibodies offer the advantages of being chemically uniform, providing high specificity, and they can be produced in large amounts. As a result, more and more researchers and manufacturers have turned to monoclonal antibodies for the development of apolipoprotein immunoassays. However, generating and selecting monoclonal antibodies that are appropriate for the measurement of apolipoproteins A–I and B are difficult and time-consuming. On the other hand, an inadequate characterization of the monoclonal antibodies can lead to technical artefacts and to no comparability of the data. In order to facilitate the appropriate use of monoclonal antibodies in the measurement of apo A–I and B, some important considerations for generating and selecting suitable monoclonal antibodies have been outlined by the IFCC's Committee on Apolipoproteins.

Correspondence to Santica Marcovina, Northwest Lipid Research Center, 2121 N 35th St, Seattle, Washington 98103, USA.

† Members of Committee on Apolipoproteins are: S. M. Marcovina (USA, Chairman); J. J. Albers (USA); G. R. Cooper (USA, until December 1989); J. C. Fruchart (France); M. Rosseneu (Belgium); and J. Shepherd (Scotland).

Many of the problems encountered in the immunoassays can be related directly to the physical and chemical properties peculiar to lipoproteins, and they can be particularly important when monoclonal antibodies are used. In contrast to other plasma proteins, lipoproteins are compelx non-covalent associations of multiple lipids and proteins. Some of the apolipoprotein antigenic determinants can be concealed by the complex protein/lipid and protein/protein interactions that occur in native lipoproteins. Even when the lipoproteins are delipidated and the apolipoproteins isolated, antigenic determinants still can be masked because the delipidated apolipoproteins readily aggregate in aqueous buffers. Conformational differences between purified apo A–I and apo A–I in native lipoproteins may account for significant differences in the expression of apo A–I antigenic determinants. A further complication has been described, which is related to the specific non-enzymatic modification of apo A–I [1,2]. This modification, which can include deamination of asparagine and glutamine residues during storage and perhaps also *in vivo*, can interfere with the binding of specific monoclonal antibodies.

In addition to the above-mentioned difficulties in the selection and characterization of monoclonal antibodies for measuring apolipoproteins, all monoclonal reagents have other peculiar properties, all monoclonal reagents have other peculiar properties that require careful consideration of specific assay parameters. Some monoclonal antibodies can be remarkably sensitive to detergents, the pH or ionic strength of the buffers and the temperature of the reaction mixture. Therefore, these assay parameters need to be established and carefully followed.

Researchers and manufacturers are therefore recommended to clearly indicate the immunochemical characteristics of the monoclonal antibodies selected for the immunoassays.

### Generation and selection of monoclonal antibodies

Because of the unique characteristics of apolipoproteins as antigens in immunoassays, this document makes specific recommendations for generating and selecting

© 1990 IFCC.

the best monoclonal antibodies for use, and for characterizing the selected antibodies. The best antibodies will usually be of high affinity. Therefore, to select high affinity monoclonal antibodies, the following procedures are recommended. First, classic immunologic dogma has demonstrated that multiple injections of low doses of antigen (10–50 μg of protein) over long period of time will result in the generation *in vivo* of a limited repertoire of antibodies of highest affinity. Therefore, a minimum of four intraperitoneal immunizations is recommended at 2 week intervals. The antigen can also be injected at several sites by subcutaneous or intradermal routes. If time permits, more immunizations over longer periods of time with larger intervals between injections are preferred. The use of adjuvants will in most cases improve the response. Second, because it is desirable to amplify *in vivo* only those plasmacells which produce immunoglobulin of the highest affinity, the intravenous injections given on days −4, −3, and −2 before fusion should contain only low doses of antigen, preferably one-third to one-half of the intraperitoneal dose. It is advisable to screen the serum of the immunized mice for reactivity against the immunizing antigen, in order to select for boosting the mouse with the highest antibody titre. It is usual to try to reach a titre (inverse of serum dilution at which the signal in the assay is decreased by 50%) no less than $10^4$. However, even though the serum titre elicited has undoubted relevance, it should be noted that a good titre after the preimmunization period is not a sufficient condition for a high specific efficiency. This is more dependent on an adequate final immunization protocol. Third, either purified apolipoprotein or isolated lipoprotein can be used for immunization. However, to ensure that the antibodies selected are capable of identifying the native lipoprotein, the hybridomas must be screened after fusion for their capacity to produce antibodies that bind the native lipoprotein. Only freshly prepared antigens should be used for immunization and screening. Fourth, to select only those antibodies that are capable of binding antigen at low concentrations, the hybridomas should always be screened at high dilution. Dilutions of the culture supernantants of 1:100 or higher are preferred, although, in this situation, a low concentration of high affinity antibody could be missed. Fifth, a secondary screen should be performed on all antibodies to assess their capacity to bind antigen in solution, as well as antigen bound to a solid-phase matrix. This assay can take a number of formats, including immobilization of the antibody or classical double antibody fluid-phase immunoassay using radioiodinated LDL or HDL. The format that is ultimately to be used for the immunoassay should also be used as the secondary screening step. Sixth, because IgG antibodies generally are preferable for use in immunoassays, screening for specific immunoglobulin heavy chains can be useful. A number of kits for identifying the mouse immunoglobulin heavy chain types are available.

### Specificity

The problem of specificity has always been a critical issue in the preparation of polyclonal antisera. When found, the lack of specificity of an antiserum was attributed to the presence of contaminants in the antigen preparation used for immunization, and the unwanted specificities were eliminated by immunoadsorption. However, totally unexpected cross-reactivities between apparently unrelated molecules have been detected with monoclonal antibodies to several proteins [3]. A series of proteins which show immunological cross-reactivity with human apo D also have been identified [4]. Presumably these antibodies exist in different amounts in polyclonal antisera and can be responsible for the lack of specificity. The most common criteria for assuring the specificity of antibodies against apolipoproteins is to verify that the antibodies react with the apolipoprotein in question and not with other purified apolipoproteins. In general these studies are performed by direct or competitive enzyme immunoassay or radioimmunoassay. These tests, however, do not exclude cross-reactivities with other molecules in plasma. To document that the monoclonal antibodies are specific for one apolipoprotein, the most discriminating test, one or two-dimensional Western immunoblotting [5–7], is recommended. Electrophoretically separated proteins are transferred to a nitrocellulose sheet for reaction with dilutions of antibody. Monoclonal antibody binding is detected by a second incubation with a radioiodinated second antibody (usually rabbit or goat anti-mouse Ig) followed by autoradiography. Enzyme conjugated anti-mouse Ig, and the appropriate substrates for colour development, can also be used. To document the lack of cross-reactivity with other apolipoproteins and plasma components not associated with lipoproteins, monoclonal antibodies should be tested by Western immunoblotting against the apolipoproteins of isolated lipoproteins and whole plasma. However, it needs to be underlined that the SDS Page step prior to Western blotting is denaturing and, therefore, some clinically useful monoclonal antibodies reacting only with nondenatured antigenic determinants could fail to be detected by immunoblotting. Monoclonal antibodies to apo B should also be tested for their reactivity with apo B-48.

It has long been accepted that, for polyclonal antisera, specificity can be a function of the sensitivity of the test used to verify the specificity. Thus, an antisera, determined to be specific by immunoelectrophoresis (sensitivity in the pg/ml range). This would be expected to be less of a problem with monoclonal antibodies. Nevertheless, one might imagine a situation in which cros-reactivities exist between two native molecules (which presumably would be detected in a radioimmunoassay), but not between the denatured molecules (and thus not detected on Western blots). Therefore, the specificity of the antibodies should be confirmed in the assay in which the antibody is to be used.

### Affinity constant

Generally, high affinity monoclonal antibodies are highly specific and less sensitive to the modifications of the immunoassay conditions. For these reasons it is important at the first stage of selection to determine the affinity constant. The recommended method for determining the affinity constant is a competitive fluid-phase radioimmunoassay. The test is performed by adding increasing amounts of apolipoprotein or isolated lipoprotein to compete with a known amount of labelled lipoprotein for a limited amount of monoclonal antibody. The displacement curves are analysed by Scatchard analysis and the affinity constant calculated and expressed as litres per mole [8–10]. A simplified approach for estimating antibody affinity has been reported by Müller [11] and it can be useful in the selection of monoclonal antibodies. We recommend that the affinity constants of monoclonal antibodies selected for use in immunoassays should be at least $10^9$ l/mol.

### Recognition of isolated and native apolipoproteins

One of the most common problems found with apolipoprotein-specific monoclonal antibodies is that many of the antibodies do not recognize the isolated delipidated apolipoprotein in the same way as they recognize the same apolipoprotein in a complex lipoprotein [4,5,12]. Furthermore, differences have been observed between an isolated lipoprotein and that same lipoprotein in plasma [13]. Therefore, any monoclonal antibody used for estimating plasma levels of an apolipoprotein must be capable of identifying the apolipoprotein in the sample, in the primary standard and in the calibrator in exactly the same manner, i.e. with the same affinity. This requirement is fundamental in competitive assays, but it is less important in non-competitive assays. Also, the antibody should detect an epitope that is expressed on all lipoproteins in which the apolipoprotein is present, and the affinity for the apolipoprotein should be independent of the physical and chemical properties of the lipoprotein in which it is found. To document that the affinity of the antibody is the same, all standards, samples and calibrators must be compared as competitors and the slopes of the competition curves compared [5]. This is performed by adding increasing amounts of apolipoprotein, isolated lipoprotein or plasma to the immunoassay in the presence of a limiting amount of antibody. If the results are plotted on a linear log scale of percentage binding versus the log of the concentration of the competitor, the slopes of the linear portions of the curves can be compared. Additionally, logit transformation of the binding data will permit an analysis of the slopes of the competitors over a greater concentration range. In addition to comparisons of samples, standards and calibrators in the assay format, it is also important to establish whether storage of the samples or standards will result in changes in binding affinity.

### Immunochemical heterogeneity of apolipoproteins

A major stumbling block to the development of immunoassays with monoclonal reagents for total plasma levels of apo A–I or apo B is the observation that neither apo A–I or apo B appear to represent immunochemically homogenous proteins in lipoprotein particles. That is, many apo B-specific as well as apo A–I-specific monoclonal antibodies have been described that do not bind 100% of LDL or 100% of HDL, respectively [4,5,7,12]. For this reason, careful screening and selection procedures must be used to select monoclonal antibodies that bind all of the antigen. A number of procedures can be adapted for assessing the capacity of a monoclonal antibody to bind 100% of antigen, and each of these assays has two properties. The ligand must be labelled (i.e. LDL or HDL must be isolated and radioiodinated) and the antibody must be present in excess in the reaction mixture. There are two recommended methods. First, the assessment of percentage binding can be examined on immunoabsorbant columns. Here large amounts of monoclonal antibody are isolated, coupled to an insoluble support, and then reacted with a limiting and known amount of labelled LDL or HDL to document that in antibody excess, all of the LDL or HDL can be bound by the antibody. The non-specific absorption of support must be carefully evaluated. A second approach is to assess percentage binding of the ligand in fluid-phase double antibody radioimmunoassays. In this approach a known and limited amount of labelled LDL or HDL is reacted with increasing concentrations of monoclonal antibody in a fluid-phase reaction. At equilibrium the bound radioiodinated lipoprotein is separated from the unbound lipoprotein by direct precipitation with optimal amounts of a second antibody, reaction with an excess of immobilized second antibody, or reaction with protein A. Correct interpretation of these assays assumes that labelled and unlabelled lipoprotein ligands behave immunochemically in an identical manner, and that true monoclonal antibody excess (relative to antigen) was obtained. Monoclonal antibodies that do not bind all of LDL or HDL are generally not appropriate for use in immunoassays for plasma levels of apo B and apo A–I. In this case, a combination of monoclonal antibodies capable of identifying all LDL or HDL can be used. However, while this document is devoted to recommendation of monoclonal antibodies for measuring total apo A–I or apo B, it should be emphasized that individual monoclonal antibodies that do not bind all of LDL or HDL could potentially be used to quantify clinically important subsets of apo A–I- and apo B-containing lipoproteins.

### Recognition of unique epitopes

In some instances it may be important to document that specific monoclonal antibodies identify unique antigenic determinants on apo A–I or apo B. If capture assays are used, it is imperative that the binding of one antibody does not interfere with the binding of the other. If the antibodies are used in immunoassays in which immunoprecipitation is required as a secondary reaction, oligoclonal mixtures of the monoclonal antibodies will be required [7,14]. In some instances it may be useful to combine monoclonal antibodies into oligoclonal mixtures in order to increase the apparent affinity of a particular reaction or to reach 100% of the binding of LDL or HDL if a single monoclonal antibody alone will not do so. In each case the antibodies used must identify unique epitopes on the apolipoprotein. This can be verified by performing antibody competition assays. The antibodies are isolated, radioiodinated, and the capacity of increasing amounts of one unlabelled monoclonal antibody to interfere with the binding of a limited amount of a second radioiodinated monoclonal antibody is assessed [15]. If the binding of the unlabelled antibody is not blocked by an excess of the first unlabelled antibody, it can be concluded that the two antibodies identify separate epitopes on the apolipoprotein.

### Polymorphism

A final caution in the characterization of monoclonal antibodies for use in estimating plasma levels of the apolipoproteins concerns the recent reports that some monoclonal antibodies identify genetic polymorphisms in apo B [16,17]. The monoclonal antibody used in these studies, MB19, identifies a common genetic polymorphism in LDL. This polymorphism is characterized by the capacity of the MB19 antibody to bind to apo B-100 from different individuals with either high or low affinity. The possibility exsits that additional genetic polymorphisms in apo B, as well as in apo A–I, will be identified with monoclonal antibodies. Therefore, it must be established that any monoclonal antibody use for estimating total plasma levels of apo B or apo A–I does not distinguish a common genetic polymorphism. To do this, the plasmas of a minimum of 100 unrelated individuals should be tested and the levels of apo A–I or B measured with the monoclonal antibody compared with the levels obtained by an independent method. Independent measurements of apo A–I and apo B can be obtained with immunoassay procedures employing polyvalent antibodies or other physical chemical estimates of apolipoprotein mass. If no systematic bias is found, it can be reasonably concluded that the monoclonal antibodies do not identify a common apolipoprotein genetic polymorphism.

### References

1. Milthorp, P., Weech, P. K., Milne, R. W. and Marcel, Y. L., *Arteriosclerosis*, **6** (1986), 285.
2. Curtiss, L. K. and Smith, R. S. Immunochemical heterogeneity of HDL. *Proceedings of the Workshop on Lipoprotein Heterogeneity*, Ed. Lippel, K. (*NIH Publication, 1987*).
3. Fox, P. C. and Siraganian, R. P., *Hybridoma*, **5** (1986), 223.
4. Marcel, Y. L., Weech, P. K., Milthorp, P., Terce, F., Vezina, C. and Milne, R. W. *Progress in Lipid Research*, **23** (1985), 169.
5. Curtiss, L. K. and Edgington, T. S., *Journal of Biological Chemistry*, **257** (1982), 15213.
6. Marcel, Y. L., Hogue, M., Weech, P. K. and Milne, R. W., *Journal of Biological Chemistry*, **256** (1984), 6952.
7. Marcovina, S., France, D., Phillips, R. and Mao, S., *Clinical Chemistry*, **31** (1985), 1654.
8. Frankel, M. E. and Gerhard, W., *Molecular Immunology*, **16** (1979), 101.
9. Milne, R. W. and Marcel, Y. L., *FEBS Letters*, **142** (1982), 97.
10. Wiklund, O., Dyer, C. A., Tsao, B. P. and Curtiss, L. K., *Journal of Biological Chemistry*, **260** (1985), 10956.
11. Müller, R., *Journal of Immunological Methods*, **34** (1980), 345.
12. Curtiss, L. L. and Edgington, T. S., *Journal of Biological Chemistry*, **260** (1985), 2982.
13. Schonfeld, G. and Krul, E. S., *Journal of Lipid Research*, **27** (1986), 583.
14. Marcovina, S., Di Cola, G. and Catapano, A. L., *Clinical Chemistry*, **32** (1986), 2155.
15. Weech, P. K., Milne, R. W, Milthorp, P. and Marcel, Y. L., *Biochimica Biophysica Acta*, **835** (1985), 390.
16. Young, S. G., Bertics, S., Curtiss, L. K., Casal, D. C., Witztum, J. L., *Proceedings of the National Academy of Sciences (USA)*, **83** (1986), 1101.
17. Young, S. G., Bertics, S. J., Scott, T. M., Dubois, B. W., Curtiss, L. K. and Witztum, J. L., *Journal of Biological Chemistry*, **261** (1986), 2995.

